# Supplementary material for: Automated Segmentation of Skin Strata in Reflectance Confocal Microscopy Depth Stacks
Source: PLoS One. 2016 Apr 18;11(4):e0153208. doi: 10.1371/journal.pone.0153208 (PMC4835045; doi:10.1371/journal.pone.0153208)

Patch Preprocessing

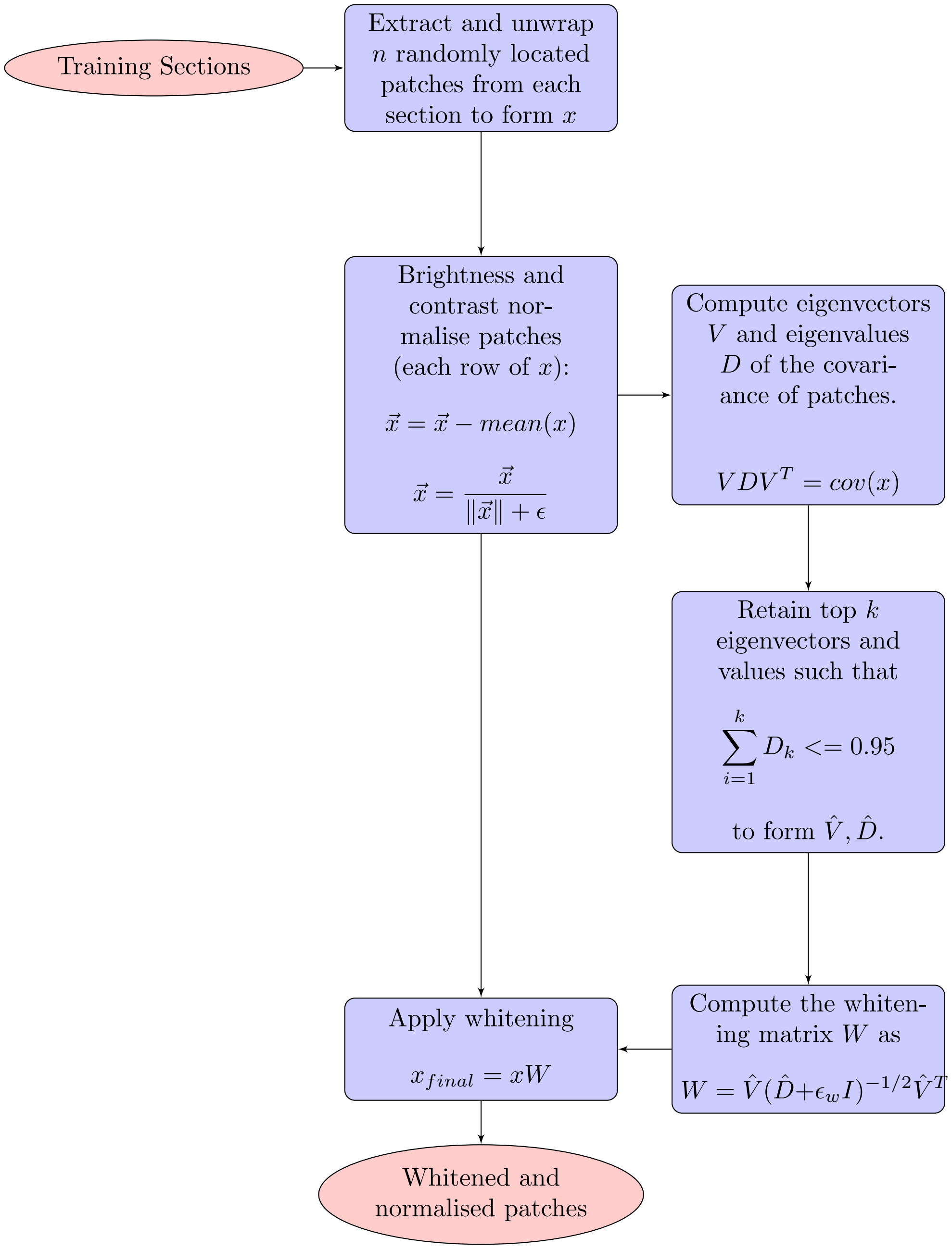

Hierarchical Spherical K-Means

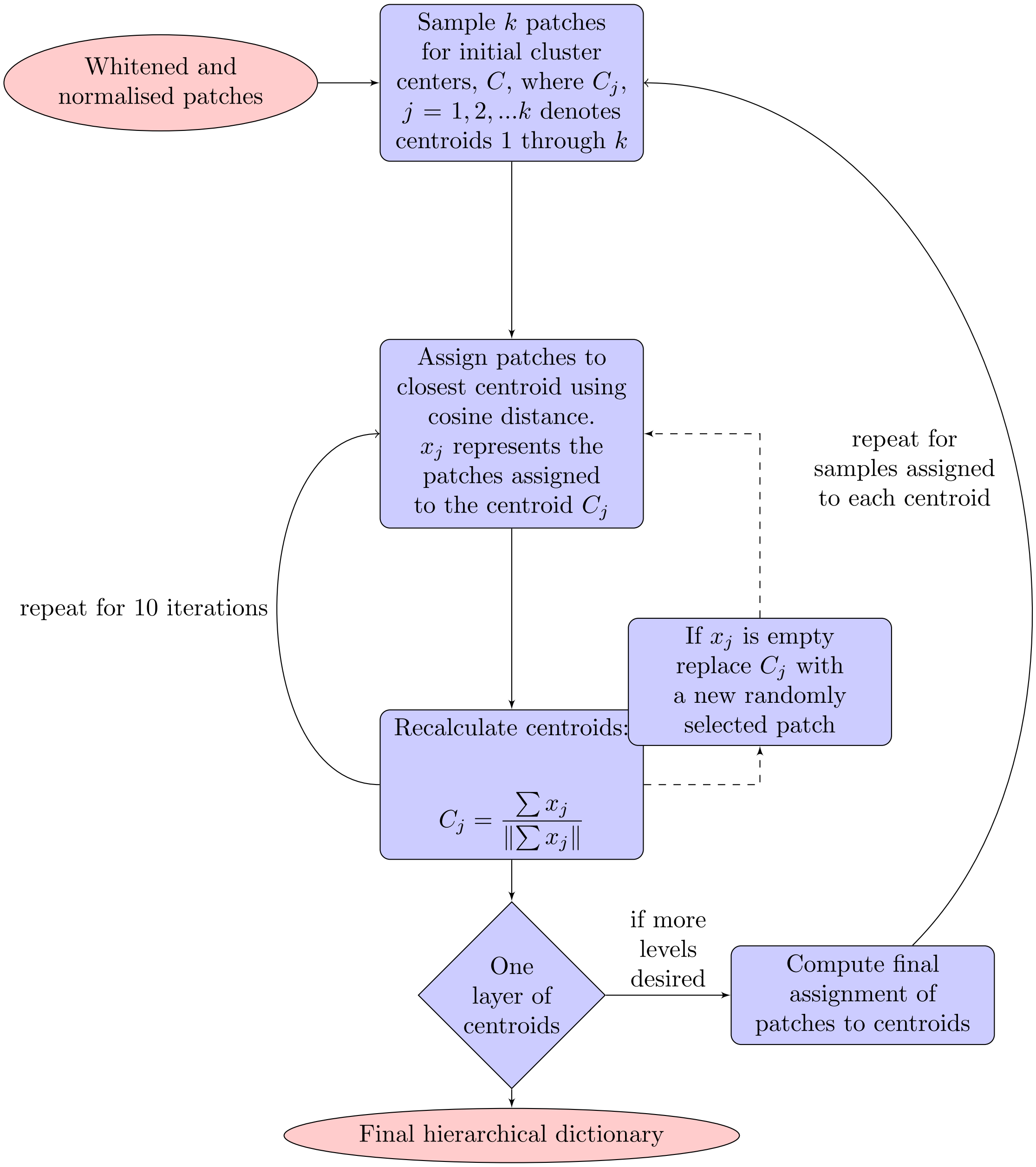

Augmentation and Section Classification

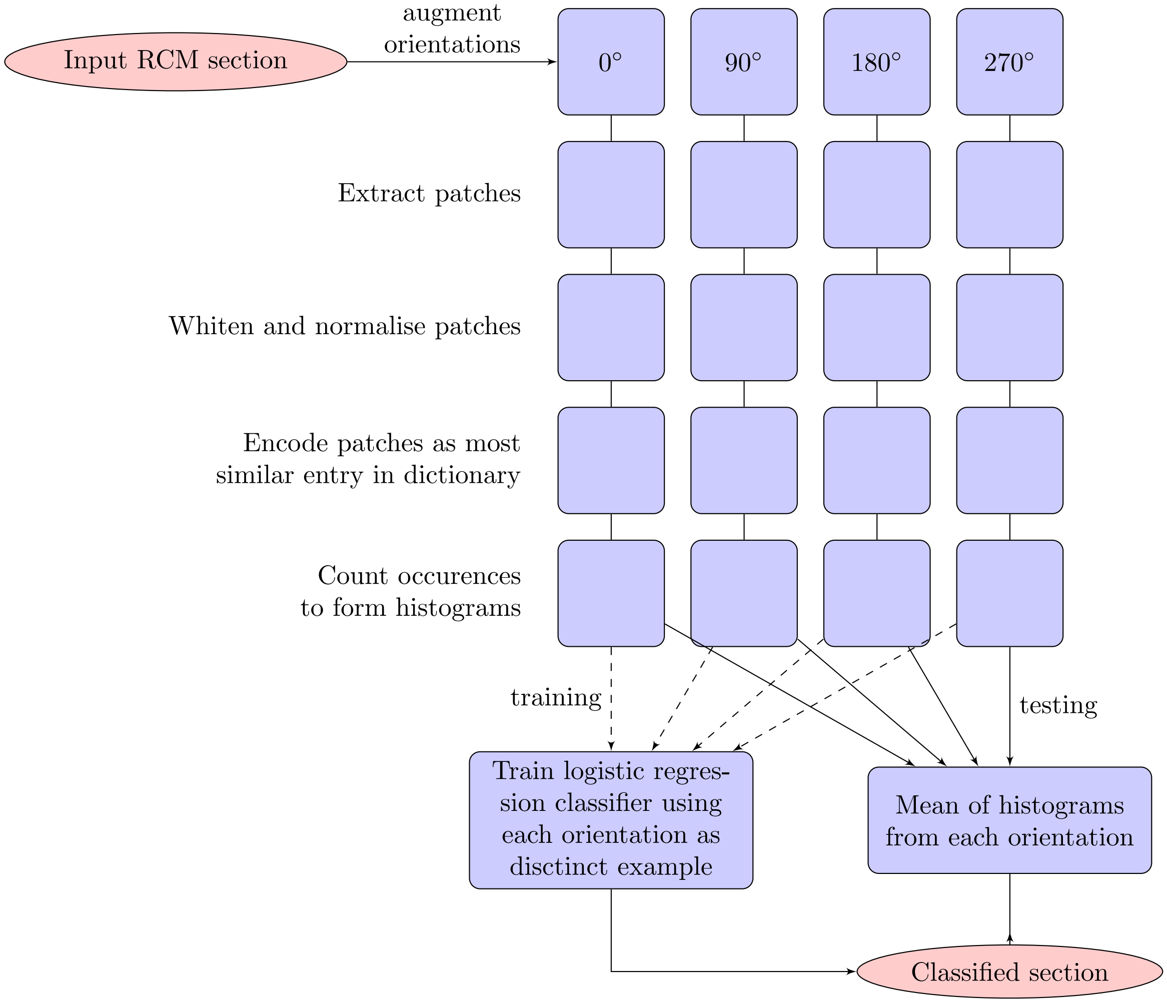

Supplement: S1 Fig — (PDF) [file pone.0153208.s002.pdf]
